# High levels of nucleotide diversity and fast decline of linkage disequilibrium in rye (*Secale cereale *L.) genes involved in frost response

**DOI:** 10.1186/1471-2229-11-6

**Published:** 2011-01-10

**Authors:** Yongle Li, Grit Haseneyer, Chris-Carolin Schön, Donna Ankerst, Viktor Korzun, Peer Wilde, Eva Bauer

**Affiliations:** 1 Technische Universität München, Plant Breeding, Freising, Germany; 2 Technische Universität München, Mathematical Statistics, Garching, Germany; 3 KWS LOCHOW GMBH, Bergen, Germany

## Abstract

**Background:**

Rye (*Secale cereale *L.) is the most frost tolerant cereal species. As an outcrossing species, rye exhibits high levels of intraspecific diversity, which makes it well-suited for allele mining in genes involved in the frost responsive network. For investigating genetic diversity and the extent of linkage disequilibrium (LD) we analyzed eleven candidate genes and 37 microsatellite markers in 201 lines from five Eastern and Middle European rye populations.

**Results:**

A total of 147 single nucleotide polymorphisms (SNPs) and nine insertion-deletion polymorphisms were found within 7,639 bp of DNA sequence from eleven candidate genes, resulting in an average SNP frequency of 1 SNP/52 bp. Nucleotide and haplotype diversity of candidate genes were high with average values *π *= 5.6 × 10^-3 ^and *Hd *= 0.59, respectively. According to an analysis of molecular variance (AMOVA), most of the genetic variation was found between individuals within populations. Haplotype frequencies varied markedly between the candidate genes. *ScCbf14*, *ScVrn1*, and *ScDhn1 *were dominated by a single haplotype, while the other 8 genes (*ScCbf2*, *ScCbf6*, *ScCbf9b*, *ScCbf11*, *ScCbf12*, *ScCbf15*, *ScIce2*, and *ScDhn3*) had a more balanced haplotype frequency distribution. Intra-genic LD decayed rapidly, within approximately 520 bp on average. Genome-wide LD based on microsatellites was low.

**Conclusions:**

The Middle European population did not differ substantially from the four Eastern European populations in terms of haplotype frequencies or in the level of nucleotide diversity. The low LD in rye compared to self-pollinating species promises a high resolution in genome-wide association mapping. SNPs discovered in the promoters or coding regions, which attribute to non-synonymous substitutions, are suitable candidates for association mapping.

## Background

Rye (*Secale cereale *L.) is a cross-pollinated cereal with a diploid genome. It is grown on approximately 6 million hectares in Europe for bread-making, animal feed, forage feeding, and vodka production (FAO, 2010). As the most frost tolerant small grain cereal [1] it is well-suited for investigations of frost tolerance. Findings in rye are of interest for less frost tolerant cereals such as wheat and barley.

Cold and frost stress, namely chilling injury at temperatures lower than 10°C and freezing injury at temperatures lower than 0°C, adversely affect plant growth and productivity via cellular damage, dehydration and metabolic reaction slow-down. A major focus of this study was to investigate candidate genes with a putative role in frost tolerance. Frost tolerance has a polygenic inheritance. Many genes involved in the cold/frost responsive network have been identified in *Arabidopsis *via quantitative trait loci (QTL) mapping, microarray analysis and transgenic expression [2,3]. These genes are mainly involved in stress signalling, transcriptional regulation, and direct response to cold/frost, including cellular membrane stabilization. The gene *Inducer of Cbf Expression 2 *(*Ice2*) is a basic helix-loop-helix transcription factor that binds to promoters of the *C-repeat Binding Factor *(*Cbf*) gene family and activates their transcription under frost stress in hexaploid wheat [4]. Over-expression of *Arabidopsis Ice2 *[5] results in increased tolerance to deep freezing stress at a temperature of -20C° after cold acclimation. The *Cbf *gene family belongs to the family of *APETALA2 *transcription factors. In barley, diploid and hexaploid wheat several cereal *Cbf *homologs have been cloned and mapped to the *Fr2 *locus on homoeologous group 5, which coincides with a major QTL for frost tolerance [6-8]. Using wheat-rye addition lines, Campoli et al. [9] assigned twelve members of the *Cbf *gene family to the long arm of chromosome 5R in rye. Several studies in *Arabidopsis *provide evidence that allelic variation in the *Cbf *gene family forms the molecular basis for the freezing tolerance QTL [10,11]. *Cbf *transcription factors activate *Cold Responsive *(*COR*) genes through binding to *cis*-elements in the promoters of *COR *genes under cold stress in *Arabidopsis *[12]. More than 70 proteins encoded by *COR *genes are involved in direct response to cold/frost. Dehydrins, also known as *Late Embryogenesis Abundant II *(*LEA II*), are among the proteins that protect other proteins and membranes from cellular damage caused by dehydration [13]. In barley, 13 dehydrin genes (*Dhn 1-13*) have been identified [14]. Transcripts of *Dhn1, Dhn2, Dhn3, Dhn4, Dhn7*, and *Dhn9 *were detected in plants subjected to cold acclimation at 4°C followed by mild frost at -2°C or -4°C [15]. *Dhn1 *and *Dhn3 *were mapped in barley to chromosome 5H near a QTL for winter hardiness and on chromosome 6H, respectively [13]. Recent studies showed that cold/frost regulation and vernalization are interconnected [16,17]. Winter cereals require long exposure to cold in winter, the so-called vernalization, to accelerate flowering in the next spring. This process prevents the early transition of winter cereals into the less cold-tolerant reproductive phase. *Vrn1 *has been mapped to the second locus conferring frost tolerance, *Fr1*, on the long arm of homoeologous group 5 near the *Fr2 *locus [18]. Transcript levels of all cold-induced *Cbf *genes at the frost tolerance locus *Fr-H2 *in barley are significantly higher in lines harbouring the *vrn1 *winter allele than in lines harbouring the *Vrn1 *spring allele [19]. It remains unknown how the *Cbf *family members interact with *Vrn1 *under frost stress.

To unveil genetic diversity among candidate genes involved in the frost response network in rye, one Middle European and four Eastern European populations were studied. Cultivated rye shows a wide range of diversity, reflecting adaptation to various environments and selection pressures [20]. Middle European populations are well-adapted to the more moderate Middle European climate which is in the transition zone between temperate and continental climate, whereas Eastern European populations show good adaptation to a continental climate with severe winters. Thus, differences between Middle and Eastern European populations in allele number and/or frequencies of frost-related candidate genes are expected. Several studies have investigated genome-wide genetic diversity in rye based on molecular markers, including isoenzymes [21] and simple sequence repeats (SSRs) [22]. None, however, have investigated locus-specific genetic diversity at the gene level.

Linkage disequilibrium (LD), the non-random combination of alleles at different loci, determines the marker density required for marker-based studies, such as association mapping or genomic selection [23]. Studies on the extent of LD in various crops, such as *Triticum durum *[24], *Zea mays *[25,26], and *Sorghum bicolor *[27], indicate large variation in the extent of LD. The effect of germplasm on LD is clearly observed in barley, where LD decays within 0.4 kb in wild material and extends up to 212 kb in elite lines [28]. LD decay can also vary considerably from locus to locus due to different recombination rates and selection pressures at different regions of the genome. In addition, higher levels of LD are observed in self-pollinating species compared to outcrossing species, indicating that mating systems play a role [23]. Since rye is an outcrossing species, a low level of LD with a rapid decay is expected. To the best of our knowledge there is no prior study on the pattern of LD within and between rye genes.

The objectives of this study were to investigate nucleotide and haplotype diversity, the extent and pattern of LD, and population differences among eleven candidate genes (*ScCbf2*, *ScCbf6*, *ScCbf9b*, *ScCbf11*, *ScCbf12*, *ScCbf14*, *ScCbf15*, *ScVrn1*, *ScIce2*, *ScDhn1*, and *ScDhn3*) involved in the frost tolerance network in five winter rye populations from Belarus, Germany and Poland.

## Methods

### Plant material and DNA extraction

Plant material was derived from five open-pollinated winter rye breeding populations, four from Eastern Europe, PR 2733 (Belarus), EKOAGRO (Poland), SMH2502 (Poland), ROM103 (Poland), and one from Middle Europe, Petkus (Germany). For convenience, they will be referred to as PR, EKO, SMH, ROM, and Petkus, respectively. The Petkus population has undergone several cycles of recurrent selection, while the breeding history of the four Eastern European populations is unknown. Since rye is an outcrossing species, it is highly heterozygous, which leads to difficulties in determining haplotype phase. To address this problem, gamete capture was performed. Between 15 and 68 heterozygous plants from each of the five populations were crossed with the self-fertile inbred line Lo152 resulting in 201 heterozygous S_0 _plants, each with one gamete known. The plants were grown in a growth chamber and DNA was extracted from leaves according to Rogowsky et al. [29].

### Candidate gene selection and primer design

Eleven candidate genes, *ScCbf2*, *ScCbf6*, *ScCbf9b*, *ScCbf11*, *ScCbf12*, *ScCbf14*, *ScCbf15*, *ScVrn1*, *ScIce2*, *ScDhn1*, and *ScDhn3*, were selected based on their association with frost tolerance in closely related species. Individual *Cbf *genes were selected based on an expression study in rye [30] and linkage mapping in barley and diploid wheat [6,8], *Vrn1 *based on linkage mapping and a real-time PCR expression study in wheat [18,31], *Ice2 *based on an expression study in wheat [4], and *Dhn1 *and *Dhn3 *based on an expression study in barley [14]. We followed the *Cbf *nomenclature proposed by Skinner et al. [32], whereby names with the same number followed by different letters describe highly identical but distinct genes, for example, the highly identical *Cbf9a *and *Cbf9b *genes first identified by Jaglo et al. [33]. Primers for all genes were designed using Primer-BLAST from the NCBI database (http://www.ncbi.nlm.nih.gov/tools/primer-blast/) based on sequences available in GenBank; information can be found in Additional file 1. Due to limited information on rye DNA sequences in GenBank, primers for *ScVrn1*, *ScIce2*, *ScDhn1 *and *ScDhn3 *were designed based on homologous genes in *H. vulgare*, *T. aestivum *and *T. monococcum*. Despite lack of homology in non-coding regions, putative functional regions of the candidate genes could be amplified. A 250 bp fragment of the promoter and first exon of *ScVrn1 *was amplified since there is evidence that this region is one of the determinants of winter/spring growth habit in barley and wheat [34,35].

### Amplification of candidate genes and DNA sequencing

Fourteen fragments of eleven candidate genes were amplified by PCR in 10 μl reaction volumes containing 10 ng DNA, 150 nM of each primer, 1x *Taq *DNA polymerase reaction buffer, 1.5 or 2.0 mM MgCl_2_, 0.2 mM of each dNTP, and 0.5 U *Taq *DNA polymerase. After an initial denaturation at 96°C for 10 min, 35 cycles were conducted at 96°C for 1 min, primer-specific annealing temperatures at 52-66°C for 1 min, 72°C for 1 min, and a final extension step at 72°C for 15 min. Details on candidate gene amplification were described in Additional file 1. The PCR products were purified in 96-well MultiScreen PCR plates (Millipore Corporation, Billerica, MA, USA) and directly sequenced through the QIAGEN sequencing service (QIAGEN, Hilden, Germany). Amplicons of each S_0 _plant were sequenced with both forward and reverse PCR primers. Sequence data were assembled into contigs and SNPs were detected using the software Variant Reporter™ V1.0 (Applied Biosystems, Foster City, CA, USA). The DNA sequence of Lo152, a homozygous inbred line, was used as the reference sequence, and alleles of this common parent were subtracted from all sequences to determine the haplotype phase. Heterozygous insertion and deletion events were detected manually by checking sequences from both strands. The web-based program Indelligent v1.2 (http://ctap.inhs.uiuc.edu/dmitriev) was used to resolve heterozygous insertion-deletion events (Indels). In case of large Indels, for example, 200 bp in *ScCbf2*, which Indelligent could not resolve, amplicons from the respective lines were sub-cloned using the TOPO TA Cloning Kit (Invitrogen, Carlsbad, CA, USA). At least five clones were sequenced to resolve heterozygous Indels. Sequences of the Lo152 reference alleles from the eleven candidate genes were submitted to GenBank under accession numbers HQ730763-HQ730773.

The actual numbers of successful PCR amplification of the 201 lines differed from gene to gene ranging from 128 lines (64%) in *ScCbf11 *to 198 (98%) in *ScVrn1*. Missing amplification products in individual lines were most likely the result of SNPs/Indels in the primer binding sites. However, absence of some *Cbf *genes in particular lines, as has recently been reported in barley and wheat [36,37] cannot be excluded as an alternative explanation.

### Sequence analysis

Sequence polymorphisms were deduced from sequence comparisons in gene-wise sequence alignments. For convenience, polymorphic sites along the sequence were numbered starting with "SNP1". Lo152 alleles were excluded from all analyses. Haplotypes and haplotype frequencies were determined within each candidate gene using DnaSP v5.10 [39] and Arlequin v3.1 [40], respectively.

Nucleotide diversity (*π*) was calculated as the average number of nucleotide differences per site between two sequences for both, the complete sequences and restricted to exons, and haplotype diversity (*Hd*) as the probability that two randomly chosen haplotypes from a given population were different [37]. Analyses of nucleotide and haplotype diversity were performed separately for each population as well as for all populations grouped together using the software DnaSP v5.10. DnaSP v5.10 does not take into account alignment gaps that may lead to underestimated diversity values. Hence, to avoid potential bias, Indels were treated as single polymorphic sites. Average nucleotide diversity (*π*) over all genes was calculated using concatenated sequences in software TASSEL v2.1 (http://www.maizegenetics.net/).

To test for selection Tajima's *D *was calculated as the difference between the mean pairwise nucleotide differences (*π*) and the number of segregating sites (*S*) relative to their standard error using the software DnaSP v5.10. The statistical significance of Tajima's *D *was obtained assuming that *D *follows the beta distribution [38]. The rate ratio of non-synonymous to synonymous substitutions (*d*_*N*_*/d*_*S*_) was calculated according to the method introduced by Yang and Nielsen [41] implemented in the program YN00 of software package PAML v4.4c [38]. Significant departure from the standard neutral model, i.e. *d*_*N*_*/d*_*S *_= 1, was assessed by the likelihood ratio test implemented in the CODEML program of PAML v4.4c.

### SSR genotyping and genetic diversity analyses

Thirty seven SSR markers were chosen based on their experimental quality and map location as providing comprehensive coverage of the rye genome. Primers and PCR conditions for rye microsatellite (RMS) and *Secale cereale *microsatellite (SCM) markers were described in detail by Khlestkina et al. [39] and Hackauf and Wehling [40], respectively. Fragments were separated using a 3130xl Genetic Analyzer (Applied Biosystems Inc., Foster City, CA, USA), and allele sizes were assigned using the program GENEMAPPER (Applied Biosystems Inc., Foster City, CA, USA). Genotyping data obtained from the SSR analyses of the 201 lines were used for the following calculations. Polymorphic information content (*PIC*) was estimated using PowerMarker v3.0 [41], and 95% confidence intervals were calculated based on 10,000 bootstrap replications. To eliminate bias whereby the observed number of alleles highly depends on the number of analysed genotypes, allelic richness (*Rs*) was estimated from a rarefaction method [42] implemented in Fstat v2.9.3 [43]. Briefly, the method estimates the expected number of alleles in a sub-sample of *n *genotypes, given that *N *genotypes have been sampled at a locus, where N ≥ n. Specifically, in this study, it was calculated as

where *N *was the number of observed genotypes (201 or less), *N*_*i *_the number of genotypes with type *i *alleles among the *N *genotypes, *n *the number of genotypes in each population, and *S *was the total number of alleles among the *N *genotypes. To visualize the degree of variation within and between populations, principal co-ordinate analysis (PCoA) was performed using NTSYSpc v2.2 (Applied Biostatistics Inc., Setauket, NY, USA) based on DICE similarity coefficients for SSRs and haplotypes of candidate genes [44]. Analysis of molecular variance (AMOVA) [45] was performed based on SSRs using Arlequin v3.1 [46] with 15,000 permutations of the data to estimate statistical significance at *P *< 0.001 for each variance component in Fisher's exact test. The Lo152 alleles were excluded from all analyses.

### Linkage disequilibrium

Linkage disequilibrium was measured by the parameter *r*^2 ^[47] for candidate genes and SSR markers using DnaSP v5.10 and TASSEL v2.1, respectively, with Indels treated as single polymorphic sites and SNPs with minor allele frequencies (MAF) < 0.05 excluded due to instability. Statistical significance of LD was calculated using Fisher's exact test [48] and decay examined exploratorily by graphs of pairwise distances (bp) versus *r*^2^. Under the mutation-drift-equilibrium model, the expected value of *r*^2 ^is

where *N *is the effective population size, and *c *is the recombination fraction between sites. With assumption of a low mutation rate and an adjustment for sample size, the expectation becomes [49]:

where Γ = 4*Nc *and *n *is the number of lines compared. The LD decay curve was estimated using a non-linear least-squares estimate of Γ fit by the *nls *function in the R software package, http://www.r-project.org, separately for each population and for all populations pooled together. The approach of Breseghello and Sorrells [50] was used to determine threshold values of *r*^2 ^that indicated significant LD. Briefly, *r*^*2 *^values were estimated from 37 unlinked SSR markers and square root transformed so that they would be better approximated by a Normal distribution. The 95th percentile from the empirical distribution of all pairwise *r *(n = 666) derived from the 37 unlinked SSR markers was selected as the threshold value, with the rationale that any values above the threshold could in high likelihood be attributable to genetic linkage. Threshold values were calculated separately for each population and for all populations pooled together. The extent of LD was estimated as the point where the LD decay curve passed below the threshold.

## Results

### DNA sequence polymorphisms

In total, 7,639 bp from eleven candidate genes in 201 rye lines were amplified resulting in 147 SNPs, nine Indels, and an average SNP frequency of 1 SNP/52 bp (Table 1). Thirty nine SNPs were non-synonymous polymorphisms resulting in amino acid replacements, 15 of which changed polarity. In the *Cbf *gene family, *ScCbf9b *had the highest number of SNPs (N = 30), of which ten were non-synonymous and three led to an exchange of amino acids of different polarity. The first intron and second exon comprising 20% of the coding sequence of *ScIce2 *were amplified, resulting in the identification of 36 SNPs, all located in the first intron. A 250 bp fragment of the promoter and first exon of *ScVrn1 *was amplified but no polymorphic site was identified, except for a 2 bp Indel. Out of nine Indels identified, seven were located in the non-coding regions of *ScCbf2*, *ScCbf9b*, *ScVrn1*, *ScDhn1*, and *ScDhn3 *and two in the coding regions of *ScCbf12 *and *ScCbf15 *without causing a frame shift (Table 1). It is noteworthy that the 200 bp Indel in the promoter of *ScCbf2 *contained two MYB and one MYC cis-elements, putative binding sites for the transcription factor *ScIce2*.

**Table 1:** Summary information of candidate gene (CG) sequences: Analyzed fragment length, gene coverage, number of lines, number of SNPs, rate ratio of non-synonymous to synonymous substitutions (*d*_*N*_*/d*_*S*_), number of Indels and haplotypes, haplotype (*Hd*) and nucleotide diversity (*π*), Tajima's *D*, and linkage disequilibrium (LD)

CG	Fragment length (bp)	**Gene coverage **^a^	**No. of lines **^**b**^	**No. of SNPs **^**c **^**(non-synonymous)**	***d***_***N***_***/d***_***S***_	No. of Indels	No. of haplotypes	*Hd *± SD	***π *± SD × 10**^**-3 **^**(only exon)**	Tajima's *D*	**Intra-genic LD (*r***^***2***^**)**
*ScCbf2*	619	5'UTR/E	169	2 (0)	0.001	1	7	0.67 **± **0.02	1.5 **± **0.1 (1.4 **± **0.1)	1.17	0.13
*ScCbf6*	495	E	197	3 (0)	0.023	0	9	0.44 **± **0.04	3.6 **± **0.3	-0.35	0.77
*ScCbf9b*	1,371	5'UTR/E/3'UTR	183	30 (10)	0.174***	1	95	0.98 **± **0.03	7.1 ± 0.3 (11.5 **± **0.2)	1.71	0.14
*ScCbf11*	623	E	128	27 (12)	0.165	0	12	0.65 **± **0.02	14.5 ± 0. 9	1.74	0.51
*ScCbf12*	754	5'UTR/E/3'UTR	141	25 (8)	0.286***	1	48	0.89 **± **0.02	8.8 ± 1.0 (7.7 **± **0.1)	0.40	0.38
*ScCbf14*	560	E	185	5 (3)	0.606**	0	4	0.17 **± **0.04	1.5 ± 0.3	-0.27	0.92
*ScCbf15*	502	E	172	3 (3)	1.490***	1	9	0.68 **± **0.04	3.0 ± 0.2	2.14*	0.30
*ScDhn1*	435	5'UTR/E	138	4 (1)	0.128**	2	12	0.33 ± 0.05	2.7 ± 0.5 (4.4 ± 0.1)	-1.86*	0.48
*ScDhn3*	514	I/E/3'UTR	130	12 (2)	0.229***	2	21	0.73 ± 0.03	8.1 ± 0.6 (8.9 ± 0.1)	0.008	0.25
*ScIce2*	1,224	I/E	189	36^d^	n.a.	0	32	0.80 ± 0.02	11.2 ± 0.6 (0)	2.34*	0.36
*ScVrn1*	542	5'UTR/E	198	0	n.a.	1	2	0.11 **± **0.03	0.4 ± 0.1 (0)	-0.33	n.a.
Total	7,639			147 (39)		9	251				

^a ^E: exon; UTR: untranslated region; I: intron

^b ^Failure of amplification in some of the lines may be due to the presence of SNPs/Indels in the binding sites of the sequences and/or the absence of some of the *Cbf *genes in some particular lines.

^c ^Minor allele frequency (MAF) > 0.05

^d ^SNPs are silent since they were all located in the first intron of the gene.

Significance levels: * *P *< 0.05, ** *P *< 0.01, *** *P *< 0.001

n.a.: not available

### Locus-wise and genome-wide genetic diversity

Nucleotide diversity (*π*) ranged from 0.4 × 10^-3 ^in *ScVrn1 *to 14.5 × 10^-3 ^in *ScCbf11*, and when restricted to exons, from 0 in *ScIce2 *and *ScVrn1 *to 14.5 × 10^-3 ^in *ScCbf11 *(Table 1). The biggest difference between analyses of *π *for the whole gene compared to restriction to exons occurred in *ScIce2 *where *π *decreased from 11.2 to 0 due to absence of SNPs in the exon. Haplotype diversity (*Hd*) ranged from 0.11 in *ScVrn1 *to 0.98 in *ScCbf9b*. A significant positive Tajima's *D *value was observed over all populations for *ScCbf15 *and *ScIce2*, whereas a significant negative value was observed in *ScDhn1*. Rate ratios of non-synonymous to synonymous substitutions (*d*_*N*_*/d*_*S*_) were < 1 for *ScCbf2*, *ScCbf6*, *ScCbf9b*, *ScCbf11*, *ScCbf12*, *ScCbf14*, *ScDhn1*, and *ScDhn3. ScCbf15 *was the only gene with a *d*_*N*_*/d*_*S *_ratio > 1. *d*_*N*_*/d*_*S *_was significant for *ScCbf9b*, *ScCbf12*, *ScCbf14*, *ScCbf15*, *ScDhn1*, and *ScDhn3*. Due to lack of polymorphisms in their coding sequences *d*_*N*_*/d*_*S *_was not calculated for *ScIce2 *and *ScVrn1*.

**Figure 1:**
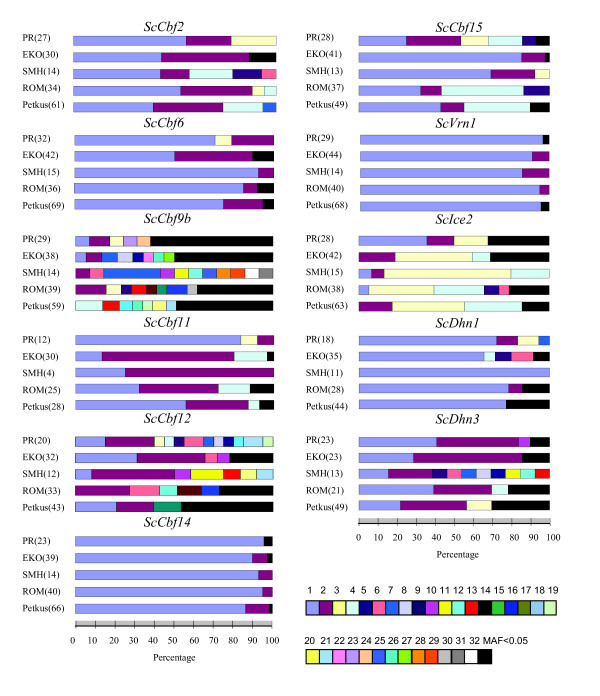
**Haplotype frequencies of eleven candidate genes in five rye populations (PR, EKO, SMH, ROM, Petkus)**. The different haplotypes occurring within each gene are represented by different coloured bars (see legend). Haplotypes occurring at a frequency < 0.05 are pooled and shown as black bars. The number of investigated lines in each population is shown in brackets.

In the SMH population, *ScCbf6*, *ScIce2*, and *ScDhn1 *had reduced nucleotide and haplotype diversities. Similarly in the PR and EKO populations, respectively, *ScCbf11 *and *ScCbf15 *had reduced nucleotide and haplotype diversities compared to the other genes (Additional file 2). Haplotype frequencies varied markedly between candidate genes, with some candidate genes dominated by a single haplotype and others with a more balanced haplotype frequency distribution (Figure 1). For example, in *ScCbf14*, *ScVrn1*, and *ScDhn1*, the most frequent haplotype occurred in more than 70% of genotypes, whereas in *ScCbf9b *all haplotypes occurred with frequencies less than 10%. The finding in *ScCbf9b *can be attributed to a large number of haplotypes (N = 95) with high haplotype diversity primarily generated by polymorphic sites located in the coding region. Similarly, only five of 48 haplotypes in *ScCbf12 *occurred at a frequency greater than 10%. For *ScCbf14*, all populations had a similar distribution of haplotype frequencies. However, for *ScCbf15 *haplotypes 1, 2, 3, and 4 were evenly distributed in PR, whereas in the other four populations only two haplotypes (EKO and SMH: 1 and 2; ROM and Petkus: 1 and 4) were prevalent (80% - 95%). For *ScCbf11*, haplotype 1 was predominant in the PR and Petkus populations, occurring in 82% and 57% of lines, respectively, whereas haplotype 2 predominated in EKO (67%) and SMH (75%).

Genetic diversity within the five populations was summarised based on 37 genome-wide SSR markers (Table 2). A total of 230 alleles and an average of 6.2 alleles per locus were observed. *PIC *varied from 0.37 ± 0.02 to 0.51 ± 001 with an average of 0.47. Allelic richness, which is not affected by sample size, ranged from 2.51 to 3.43, with a mean of 3.16. *PIC *was highly correlated with allelic richness (*r *= 0.965). Compared to the four Eastern European populations, the Petkus population had a slightly lower mean number of alleles per locus, PIC, allelic richness and number of private alleles, despite the fact that it had the largest population size. Genetic diversities of individual SSR markers across the five populations are provided in Additional file 3.

**Table 2:** Genetic diversity within populations based on 37 SSR markers

Population	No. of lines	**No. of private alleles **^**a **^**(%)**	Average no. of alleles (range)	**PIC **^**b **^**± SD**	**Allelic richness **^**c**^
PR	33	20 (12.1%)	4.46 (2-12)	0.50 ± 0.02	3.43
EKO	44	14 (8.8%)	4.30 (2-18)	0.49 ± 0.03	3.28
SMH	15	3 (2.4%)	3.38 (1-9)	0.46 ± 0.03	3.18
ROM	41	13 (7.7%)	4.50 (2-13)	0.51 ± 0.01	3.38
Petkus	68	4 (3.6%)	3.00 (1-10)	0.37 ± 0.02	2.51
Mean		10.80	3.93	0.47	3.16

^a ^Private alleles denotes the number of alleles which occurred only in one population.

^b ^PIC^: ^Polymorphic information content, a higher value means higher genetic diversity.

^c ^Allelic richness is a measure of the number of alleles independent of sample size, a higher value means higher genetic diversity.

### Genetic variation within and between populations

**Figure 2:**
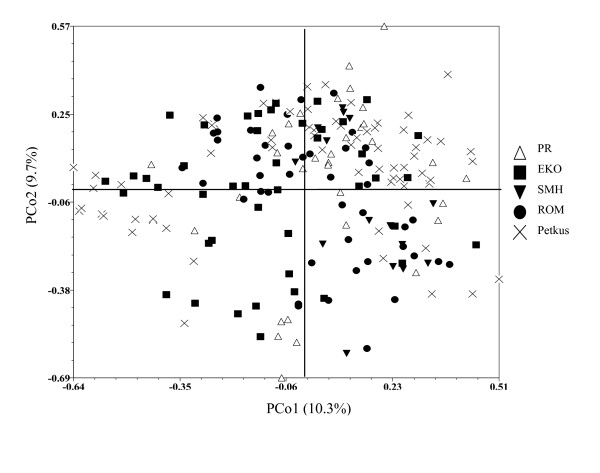
**Principal co-ordinate analysis of 201 rye lines from five populations (PR, EKO, SMH, ROM, Petkus) based on candidate gene haplotypes**. Analysis was based on a similarity matrix of candidate gene haplotypes. PCo1 and PCo2 are the first and second principal co-ordinates and percentages indicate percent variation explained.

**Figure 3:**
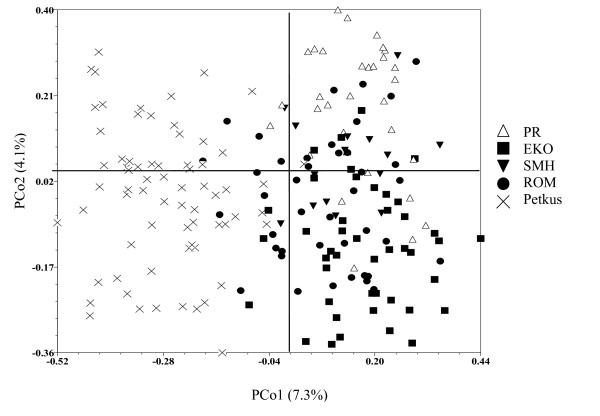
**Principal co-ordinate analysis of 201 rye lines from five populations (PR, EKO, SMH, ROM, Petkus) based on genome-wide SSR markers**. Analysis was based on a similarity matrix from 37 SSR loci. PCo1 and PCo2 are the first and second principal co-ordinates and percentages indicate percent variation explained.

PCoA of candidate gene haplotypes revealed large genetic variation within each population and no clustering according to population membership (Figure 2). The first and second principal co-ordinates explained 10.3% and 9.7% of the total genetic variation, respectively. PCoA of the 37 genome-wide SSRs similarly identified most genetic variation as residing within populations (Figure 3). However, it could differentiate the Petkus population from all Eastern European populations, and the PR population from the other three Eastern European ones. The first and second principal co-ordinates explained 7.3% and 4.1% of the total genetic variation, respectively. AMOVA revealed low variation (13.3%) between populations, but high variation (86.7%) within populations (Additional file 4).

### Linkage disequilibrium

**Figure 4:**
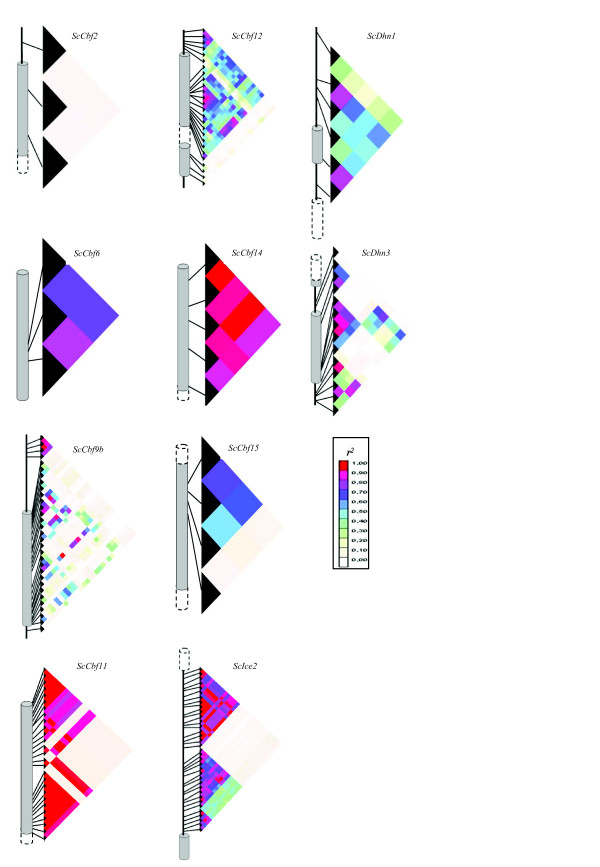
**LD heat plots of ten candidate genes**. Analysed sequences, including the promoter and complete coding sequences of *ScCbf6 *and *ScCbf9b*, and partial coding sequences of *ScCbf12*, *ScCbf14*, and *ScCbf15*; *ScVrn1 *was not included due to a lack of pairwise comparisons, since only one Indel was observed. Exons, and 5'- or 3'-flanking regions are represented by grey cylinders and black lines, respectively. White cylinders with dashed lines indicate non-amplified exons. Black triangles represent polymorphic sites starting from "SNP1" on the top of each graph. Each grid represents the strength of LD estimated by *r^2 ^*for each pairwise comparison between polymorphic sites with a minor allele frequency (MAF) > 0.05. The colour legend for *r*^*2 *^values is given on the right side.

**Figure 5:**
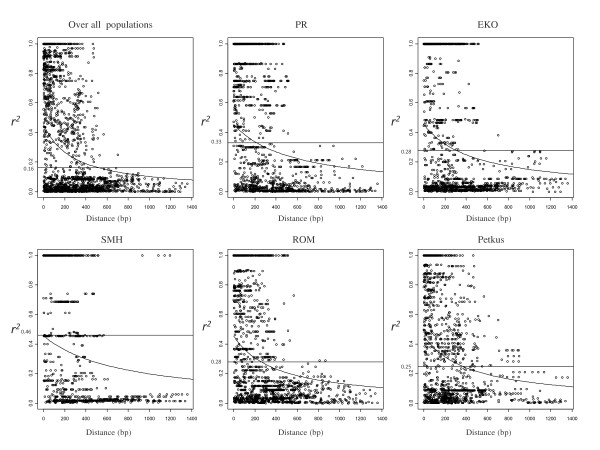
**Scatterplots of pairwise distances and LD**. LD based on *r*^2 ^between all SNPs (MAF > 5%) in eleven candidate genes within five rye populations (PR, EKO, SMH, ROM, Petkus) and across populations (over all), with non-linear fitting curve from the mutation-recombination-drift model (see methods). Thresholds for LD (see methods) are indicated by a horizontal solid line.

The mean *r*^*2 *^for pairs of SNPs within candidate genes ranged from 0.13 to 0.92 (Table 1). Two strong LD blocks were observed, one in the coding sequence of *ScCbf14 *and one in the promoter region of *ScCbf9b*, with mean *r*^*2 *^values 0.92 and 0.85 within the two LD blocks, respectively (Figure 4). In *ScCbf11*, two strong LD blocks were observed, one in the interval from SNP1 to SNP12 spanning 99 bp (mean *r*^*2 *^within LD block = 0.93), and one from SNP17 to SNP27, spanning 243 bp (mean *r*^*2 *^within LD block = 0.98). On the contrary, low LD was observed in *ScCbf2 *(mean *r*^*2 *^= 0.13), *ScDhn3 *(mean *r*^*2 *^= 0.25) and in the coding sequence of *ScCbf9b *(mean *r*^*2 *^= 0.14). Estimation of LD in *ScIce2 *was performed based on 36 SNPs (mean *r*^*2 *^= 0.36), all located in the first intron of the gene. There were three strong LD blocks, from SNP1 to SNP18 (block 1), SNP19 to SNP31 (block 2), and SNP32 to SNP36 (block 3), spanning 458 bp, 187 bp, and 61 bp, with a mean *r*^*2 *^within LD blocks of 0.85, 0.75, and 0.73, respectively. Interestingly, the mean *r*^*2 *^between blocks 2 and 3 decreased to 0.35, between blocks 1 and 2, further to 0.10, and between blocks 1 and 3, to 0.13. The inter-genic LD among the *ScCbf *genes was very low (mean *r*^*2 *^= 0.05), and only *ScCbf14 *showed a slightly higher LD (mean *r*^*2 *^= 0.15) than *ScCbf9b *(data not shown). Threshold values of *r*^2 ^as determined from 37 unlinked SSR markers varied from 0.16 over all populations to 0.46 in the SMH population. The average extent of significant LD pooling all candidate genes and populations together was approximately 520 bp (Figure 5). There were 2,194 pairwise comparisons of polymorphic sites, of which almost one third were significant as determined by Fisher's exact test. The average extent of significant LD in individual populations was much smaller because of more stringent threshold values and ranged from 0 to approximately 380 bp in the SMH and Petkus populations, respectively. Extent of LD ranged from approximately 80 bp in *ScCbf15 *to 800 bp in *ScIce2 *(Additional file 5). In *ScCbf11*, *ScCbf14*, and *ScDhn1*, mean *r*^*2 *^remained larger than 0.16 within the 400 bp amplified region. As expected LD based on genome-wide SSR markers was low with a mean *r*^*2 *^= 0.01 (data not shown).

## Discussion

### High level of nucleotide and haplotype diversity in rye

We investigated the genetic diversity of five winter rye populations from Middle and Eastern Europe. SNP frequency and nucleotide diversity are affected by several factors, including selection, mutation, mating system, effective population size, and demography [51]. SNP frequency observed in the 5 rye populations under study was on average 1 SNP every 52 bp and the average nucleotide diversity (*π*) ranged from 0.4 × 10^-3 ^to 14.5 × 10^-3 ^with an average value of *π *= 5.6 × 10^-3^. These values are as high as those reported in maize landraces, where one study reported a rate of one SNP per 62 bp, a range of *π *from 0.1 × 10^-3 ^to 13.3 × 10^-3 ^and an average value of *π *equal to 4.0 × 10^-3 ^[52]. Some studies have suggested that comparisons among different species should be restricted to homologous genes [53]. Nucleotide diversities of three *Cbf *homologs (*AtCbf1, AtCbf2 *and *AtCbf3*) in 34 *Arabidopsis *ecotypes ranged from *π *= 2.6 × 10^-3 ^to 6.9 × 10^-3 ^[54], a smaller range compared to this study (*π *= 1.5 × 10^-3 ^to 14.5 × 10^-3^), which is likely due to the different mating system. In addition, the *Cbf *gene family in rye encompasses more members than in *Arabidopsis*, which could result in less selection pressure on individual genes with complementary function in the frost tolerance network and consequently in a higher nucleotide diversity. The buffering effect induced by a large number of duplicated genes leads to a higher variation in individual duplicated genes, a phenomenon also observed in polyploid plants [55]. It is worth re-iterating that inference concerning the nucleotide diversity of *ScVrn1 *was restrained since only a partial fragment of the gene, 30% of the coding region, could be amplified due to limited available rye sequences for primer design. Observed haplotype diversities of *HvCbf9b *in *Hordeum spontaneum*, old cultivars and modern cultivars of *H. vulgare *were 0.48, 0.18, and 0.06, respectively, which is much lower than that of *ScCbf9b *in this study (0.98 ± 0.03) [36].

### Directional selection

A reduced genetic diversity was observed in five of the eleven genes. One possible explanation is that directional selection on the loci responsible for fitness related traits such as frost tolerance might reduce diversity within locally adapted populations due to an increase in the frequency of alleles contributing to adaptation [56]. *ScCbf15 *and *ScIce2 *showed significant positive values of Tajima's *D *(2.14 and 2.34, respectively; *P *< 0.05) over all populations, indicating balancing selection, whereby genotypes carrying alleles with intermediate frequency are favored. Positive Tajima's *D *values can also be observed if a population was formed from a recent admixture of two different populations, which cannot be excluded in this study. *Dhn1 *showed a significant negative value of Tajima's *D *(*P *< 0.05), indicating purifying selection, whereby an excess of polymorphisms with low frequencies was observed. However, population growth can also result in significant negative values of Tajima's *D*. Interestingly, *Dhn1 *in Scots pine has also been described as subject to positive selection [57], implying that *Dhn1 *is possibly a target of selection in different species. *ScCbf9b*, *ScCbf12*, *ScCbf14*, *ScDhn1*, and *ScDhn3 *had a *d*_*N*_*/d*_*S *_ratio significantly smaller than 1 (*P *< 0.01 or *P *< 0.001), whereas *ScCbf15 *had a *d*_*N*_*/d*_*S *_ratio significantly greater than 1 (*P *< 0.001). These findings can be interpreted as indication for purifying and positive selection, respectively [58]. However, it was pointed out that inferring selection pressure based on the *d*_*N*_*/d*_*S *_ratio is difficult from within-species data where segregating polymorphisms rather than fixed substitutions are observed [53,58,59].

Based on haplotype frequencies in the eleven candidate genes, the single Middle European population did not distinctly differ from the Eastern European ones (Figure 1). Since we have no information on the temporal breeding history of the Eastern European populations, it is beyond the scope of this study to make inferences on the selection pressure due to contrasting winter temperatures in these Middle and Eastern European populations. One possible explanation for a lack of differentiation might be seed exchanges between them. However, little is known about these processes, since pedigrees of the four Eastern European populations were not accessible.

### Genetic variation within and among populations

Assessment of genetic diversity based on genome-wide SSRs and locus-specific candidate genes are complementary investigations, the former providing a global view of the rye genome and the latter restricted to genes involved in the frost tolerance network. Genome-wide assessment of diversity using SSR markers revealed a higher genetic diversity for the Eastern European populations PR, EKO, SMH, and ROM compared to the Middle European Petkus population. One reason for this finding might be a bottleneck effect due to a higher selection pressure in the Petkus population, whereby it could be assumed that many "unfavourable" minor alleles were eliminated to pave the way for plants with desirable traits. The Petkus population, one of the major heterotic groups in rye, has systematically been improved by more than 5 cycles of full sib recurrent selection and a reduction in allele diversity of SSR markers due to hitchhiking with linked loci which were targets of selection is probable. The reduction of genetic diversity due to human-induced selection has been well documented in barley and maize [36,60,61]. By contrast, the Eastern European populations experienced a lower selection pressure by mass or half sib selection in the breeding programs, where introgression of foreign material was common in order to keep genetic variability on a high level. Interestingly, no reduction of genetic diversity was observed in the Petkus population based on candidate genes. One possible explanation is that at the time where selection took place, winters in Germany, the provenance of the Petkus population, were harsh enough to form a similar selection pressure on the Petkus population compared to Eastern European populations under Eastern European winters. It must be stated however, that Petkus is the only representative for the Middle European rye populations in our study and thus our conclusions on population differences must be limited to the Petkus population. Another reason could be that frost tolerance is a complex quantitative trait involving large gene networks comprising individual genes contributing only small effects, thereby making it difficult to detect selection signatures, such as reduction of genetic diversity in candidate genes. PCoA based on both candidate genes and SSRs showed high genetic variation between individuals within populations and limited clustering of lines from the same population, findings in accordance with previously reported investigations of 26 rye populations based on isoenzyme markers [21] and 12 rye populations based on RFLP markers [21]. Similar results have also been reported in other outcrossing species, including white clover [62] and perennial ryegrass [63], probably a consequence of the obligate cross-pollinated reproductive behaviour of outcrossing species. On the contrary, investigations in the self-pollinated species rice have revealed larger variation between populations [64].

### Rapid decay of linkage disequilibrium in rye

The extent of LD in rye across all eleven candidate genes and over all populations was approximately 520 bp using *r*^*2 *^= 0.16 as a critical threshold estimated from a separate analysis of 37 unlinked SSR markers. This rapid decay of LD could be expected, because compared to self-pollinated species, cross-pollinated rye has a higher effective recombination rate, which leads to a rapid decay of LD [23]. LD decays rapidly in other cross-pollinated species, including douglas fir, maize and ryegrass [25,53,65]. However, in self-pollinated species LD can extend up to 10-30 kb as in *Arabidopsis *[66,67] and 212 kb in cultivated barley [28]. Pairwise LD measured by *r*^2 ^based on SSRs was very low (mean *r*^*2 *^= 0.01), which was expected since the 37 SSRs have an average marker interval of 21 cM according to the integrated consensus map of Gustafson et al. [68].

LD results from the interplay of many factors. Selection, which causes locus-specific bottlenecks, is one of the factors that increases LD between selected alleles at linked loci. Homologs of *ScCbf *(except *ScCbf11 *in this case) were closely linked and located in the *Fr-H2*/*Fr-A*^*m*^*2 *frost locus spanning approximately 0.8 cM in the genetic maps of barley and diploid wheat on homoeologous group 5 [6,8,17]. The order of *Cbf *genes in the genetic map is consistent in both species [17]. The *Cbf *gene family is a large regulatory gene family with more than 20 members in barley, diploid and hexaploid wheat [6,8,69], sharing a high sequence similarity and induced under frost stress. It has been suggested that the members of the *Cbf *gene family have slightly different functions in the frost responsive network [8,9]. In this study, a large variation of mean *r*^*2 *^in seven *Cbf *genes (0.13 to 0.92) was observed, indicating that the family has probably undergone diverse selection history. LD can be increased by selection, for instance, by selective sweeps in which the alleles at flanking loci of a locus under selection are rapidly swept to high frequency or fixation [70]. *Arabidopsis' AtCbf2 *was implicated as subject to selection, resulting in functional divergence from *AtCbf1 *and *AtCbf3 *after *Cbf *gene duplication [54]. In this study an observed strong LD block and low nucleotide diversity in *ScCbf14 *indicated a selective sweep. Among *Cbf *family members, *TaCbf14 *has been mapped to the highest peak of the frost tolerance QTL in hexaploid wheat [7]. Two of the SNPs in *HvCbf14 *were statistically associated with frost tolerance in a European germplasm collection of spring and winter barley [36]. It remains to be demonstrated that the LD block of *ScCbf14 *found in this study has an influence on frost tolerance in rye.

## Conclusions

Genetic diversity is vital to crop improvement. This study of eleven candidate genes with a putative role in frost response and 37 genome-wide SSRs demonstrated high genetic diversity among five winter rye populations from Middle and Eastern Europe. Most of the diversity was observed within populations. The Middle European Petkus population differed neither in terms of haplotype frequencies nor in nucleotide diversities in eleven candidate genes from the four Eastern European populations. LD within candidate genes decayed rapidly, falling below *r*^*2 *^= 0.16 within approximately 520 bp. In contrast to selfing species, such as *Arabidopsis *or barley, low LD in rye promises a higher resolution in genome-wide association mapping. A challenge, however, is that many more markers are required for covering the whole genome. Given the huge genome size of rye, (~8,100 Mb) and until high-density genotyping arrays for rye become available, candidate gene based association mapping remains the most appropriate strategy for gene identification. The SNPs discovered in the promoter or coding regions of the genes investigated in this study, which cause non-synonymous substitutions, are suitable candidates for association mapping and will be studied in more detail with respect to their role in the expression of frost tolerance in rye.

## Authors' contributions

YL carried out the candidate gene and statistical analyses and drafted the manuscript. GH participated in the molecular and statistical analyses. DA provided advice for the statistical analysis. VK provided SSR marker data. PW developed the plant material. EB, CCS, PW, and VK conceived the study. All authors read, edited and approved the final manuscript.

## Supplementary Material

Additional file 1 **Primer information and details on PCR amplification of eleven candidate genes**. Click here for file

Additional file 2 **Genetic diversities of eleven candidate genes within five rye populations**. Click here for file

Additional file 3 **Chromosomal locations and diversities of the 37 SSRs**. Click here for file

Additional file 4 **Analysis of molecular variance (AMOVA) based on 37 SSR markers**. Click here for file

Additional file 5 **Scatterplots of pairwise distances and LD**. Click here for file
