# Supplementary material for: High levels of nucleotide diversity and fast decline of linkage disequilibrium in rye (Secale cereale L.) genes involved in frost response
Source: BMC Plant Biol. 2011 Jan 10;11:6. doi: 10.1186/1471-2229-11-6 (PMC3032657; doi:10.1186/1471-2229-11-6)
Supplement: Additional file 1 — Primer information and details on PCR amplification of eleven candidate genes. [file 1471-2229-11-6-S1.PDF]

Additional file 1: Primer information and details on PCR amplification of eleven candidate genes.

Primer set name	Forward (F) and reverse (R) primer sequence (5'-3')	PCR product size (bp)	Sequence for primer design (GenBank accession number)	Annealing temperature (°C)	*Taq* DNA polymerase supplier [d]	Additive	Final $MgCl_2$ concentration
ScCbf2	F: CCTCGATCGGCCGGCGTGTAGC<br>R: GTCCATGCCGCCGATCCAGTGCTC	700-900 [c]	*T. monococcum* (AY951945)	66	QIAGEN	-	1.5 mM
ScCbf6	F: ATGTGTCCGATCAAGAGGGA<br>R: CTAGCTCTGGTAGCTCCAGA	700	*S. cereale* (EU194242)	60	QIAGEN	Q-solution	1.5 mM
***ScCbf9b*-1** [a]	F: TCTAGTGGTTGACGTGTGGG<br>R: CGTCTCGTGGAACTTGGTC	770	*T. monococcum* (AY951945)	62	GE Healthcare	-	1.5 mM
***ScCbf9b*-2** [b]	F: ACCACTACTCCACACCTCTCACGA<br>R: TCCCCCAAAAGTAGAAACC	950	*S. cereale* (AF370730)	56	GE Healthcare	-	1.5 mM
ScCbf11	F: ATGGAGTGGGCGTACAGCGG<br>R: GTCAGTAGTTCCACAGGCTGA	620	*S. cereale* (EU194240)	63	QIAGEN	-	1.5 mM
***ScCbf12*-1**	F: GCCTCAACTTCCCGGACT<br>R: TCTTTCTTGTTTGCCAGCCT	600	*T. aestivum* (EF028763)	52	GE Healthcare	-	1.5 mM
***ScCbf12*-2**	F: GCGTCCCGCAAAACTATAAA<br>R: ATGTCGTGGCACAATGAGTC	700	*T. aestivum* (EF028763)	63	QIAGEN	-	2.0 mM
ScCbf14	F: GTGATGGGCACAGGACG<br>R: TTTCACAATGAACGAGCACG	720	*T. monococcum* (AY951945)	65	GE Healthcare	-	1.5 mM
ScCbf15	F: AGCTCTCCTTCCTCTCCGTC<br>R: GCCTTCAGTGTCCCAGCAC	600	*T. aestivum* (EF028765)	64	GE Healthcare	-	1.5 mM
ScDhn1	F: CCACGTAGCACGCACGCTGT<br>R: TCTTCCTCCTCCCGCCCACG	550	*H. vulgare* (AF043087)	61	QIAGEN	-	1.5 mM
ScDhn3	F: TGGTGGGCATTTCCAGCCCG<br>R: ACGTCCCGGGTACATACAAGCA	700	*H. vulgare* (AF043089)	61	QIAGEN	-	1.5 mM
***ScIce2*-1**	F: GCACTTGATGGTGAATTTTGG<br>R: TGATTGCGAACAAAAGCAAG	780	*T. aestivum* (EU562184)	62	GE Healthcare	-	1.5 mM
***ScIce2*-2**	F: TCCCTTCTCAGCTTGTTGAA<br>R: GAGGAAGCTATTGGCTGTCG	800	*H. vulgare* (DQ113909)	62	GE Healthcare	-	1.5 mM
ScVrn1	F: GGAGATTCGCACGTACGAT<br>R: ATGACTCGGTGGAGAACTCG	600	*T. monococcum* (AY188331)	58	GE Healthcare	-	1.5 mM

[a] Fragment 1
[b] Fragment 2
[c] Contains 200 bp Indel
[d] Product order numbers: QIAGEN # 201205 (QIAGEN, Hilden, Germany), GE Healthcare # 27-0799-06 (GE Healthcare, Munich, Germany)
