# Supplementary material for: High levels of nucleotide diversity and fast decline of linkage disequilibrium in rye (Secale cereale L.) genes involved in frost response
Source: BMC Plant Biol. 2011 Jan 10;11:6. doi: 10.1186/1471-2229-11-6 (PMC3032657; doi:10.1186/1471-2229-11-6)
Supplement: Additional file 2 — Genetic diversities of eleven candidate genes within five rye populations. [file 1471-2229-11-6-S2.PDF]

Additional file 2: Genetic diversities of eleven candidate genes within five rye populations.

	PR	EKO	SMH	ROM	Petkus
ScCbf2					
No. of genotypes	27	30	14	34	61
No. of polymorphisms	2	3	3	3	3
No. of haplotypes (private)	3(0)	6(0)	5(0)	4(0)	4(1)
Hd ± SD	0.62 ± 0.07	0.64 ± 0.05	0.78 ± 0.09	0.61 ± 0.06	0.70 ± 0.03
π ± SD (×10^{-3})	1.4 ± 0. 2	1.3 ± 0.2	2.1 ± 0.4	1.2 ± 0.2	1.6 ± 0.1
ScCbf6					
No. of genotypes	32	42	15	36	69
No. of polymorphisms	3	5	3	5	3
No. of haplotypes (private)	3(0)	7(2)	2(0)	6(2)	4(0)
Hd ± SD	0.49 ± 0.09	0.62 ± 0.05	0.13 ± 0.11	0.31 ± 0.10	0.40 ± 0.06
π ± SD (×10^{-3})	3.9 ± 0.6	5.0 ± 0.3	1.2 ± 0.1	2.1 ± 0.8	3.4 ± 0.5
ScCbf9b					
No. of genotypes	29	38	14	39	59
No. of polymorphisms	25	31	27	32	30
No. of haplotypes (private)	23(12)	27(17)	11(5)	23(15)	33(26)
Hd ± SD	0.98 ± 0.01	0.98 ± 0.01	0.93 ± 0.06	0.96 ± 0.02	0.96 ± 0.01
π ± SD (×10^{-3})	6.1 ± 0.6	6.6 ± 0.6	6.8 ± 0.7	6.8 ± 0.6	6.9 ± 0.6
ScCbf11					
No. of genotypes	12	30	4	25	54
No. of polymorphisms	15	28	13	28	28
No. of haplotypes (private)	3(1)	4(1)	2(0)	6(3)	7(4)
Hd ± SD	0.32 ± 0.16	0.53 ± 0.09	0.50 ± 0.27	0.73 ± 0.06	0.60 ± 0.05
π ± SD (×10^{-3})	4.0 ± 2.8	11.5 ± 2.6	10.4 ± 5.6	16.6 ± 2.2	12.7 ± 1.5
ScCbf12					
No. of genotypes	20	32	12	33	43
No. of polymorphisms	25	23	22	24	25
No. of haplotypes (private)	12(8)	11(4)	7(3)	14(8)	21(15)
Hd ± SD	0.92 ± 0.04	0.79 ± 0.05	0.83 ± 0.1	0.89 ± 0.04	0.91 ± 0.03
π ± SD (×10^{-3})	11.7 ± 2.7	4.8 ± 2.0	6.3 ± 2.9	5.4 ± 1.3	12.7 ± 1.5
ScCbf14					
No. of genotypes	23	39	14	40	66
No. of polymorphisms	5	5	5	5	5
No. of haplotypes (private)	2(0)	3(1)	2(0)	2(0)	3(1)
Hd ± SD	0.09 ± 0.08	0.19 ± 0.08	0.14 ± 0.12	0.10 ± 0.06	0.24 ± 0.06
π ± SD (×10^{-3})	0.8 ± 0.7	1.6 ± 0.7	1.3 ± 11	0.9 ± 0.6	2.1 ± 0.5

	PR	EKO	SMH	ROM	Petkus
ScCbf15					
No. of genotypes	28	41	13	37	49
No. of polymorphisms	4	2	2	4	4
No. of haplotypes (private)	7(2)	3(0)	3(0)	4(0)	6(2)
Hd ± SD	0.83 ± 0.04	0.26 ± 0.08	0.50 ± 0.14	0.70 ± 0.04	0.69 ± 0.04
π ± SD ($\times10^{-3}$)	3.5 ± 0.3	0.5 ± 0.3	1.1 ± 0.3	3.4± 0.2	3.3 ± 0.2
ScDhn1					
No. of genotypes	18	35	11	28	44
No. of polymorphisms	5	6	0	6	6
No. of haplotypes (private)	4(1)	7(2)	1(0)	6(3)	10(7)
Hd ± SD	0.48 ± 0.13	0.56 ± 0.09	0	0.39 ± 0.12	0.41 ± 0.09
π ± SD ($\times10^{-3}$)	10.8 ± 4.4	6.0 ± 1.7	0	2.2 ± 1.0	5.8 ± 2.0
ScDhn3					
No. of genotypes	23	23	13	21	49
No. of polymorphisms	12	7	10	8	4
No. of haplotypes (private)	10(6)	8(1)	10(6)	5(1)	7(4)
Hd ± SD	0.84 ± 0.06	0.77 ± 0.06	0.95 ± 0.05	0.61 ± 0.09	0.66 ± 0.04
π ± SD ($\times10^{-3}$)	14.2 ± 1.6	7.5 ± 1.6	14.1 ± 2.0	10.2 ± 2.5	5.8 ± 0.5
ScIce2					
No. of genotypes	28	42	15	38	63
No. of polymorphisms	19	29	16	28	7
No. of haplotypes (private)	12(8)	13(9)	4(0)	13(7)	11(6)
Hd ± SD	0.84 ± 0.05	0.80 ± 0.05	0.54 ± 0.13	0.82 ± 0.05	0.74 ± 0.03
π ± SD ($\times10^{-3}$)	12.5 ± 1.3	8.7 ± 1.4	8.0 ± 1.0	12.0 ± 1.1	8.4 ± 0.5
ScVrn1					
No. of genotypes	29	44	14	40	68
No. of polymorphisms	1	1	1	1	1
No. of haplotypes (private)	2(0)	2(0)	2(0)	2(0)	2(0)
Hd ± SD	0.11 ± 0.03	0.17± 0.07	0.26 ± 0.14	0.10 ± 0.06	0.09 ± 0.05
π ± SD ($\times10^{-3}$)	0.4 ± 0.1	0.6 ± 0.2	0.9 ± 0.4	0.3 ± 0.2	0.3 ± 0.2

n.a: not available
