# Supplementary material for: High levels of nucleotide diversity and fast decline of linkage disequilibrium in rye (Secale cereale L.) genes involved in frost response
Source: BMC Plant Biol. 2011 Jan 10;11:6. doi: 10.1186/1471-2229-11-6 (PMC3032657; doi:10.1186/1471-2229-11-6)
Supplement: Additional file 3 — Chromosomal locations and diversities of the 37 SSRs. [file 1471-2229-11-6-S3.PDF]

Additional file 3: Chromosomal locations and diversities of the 37 SSRs.

SSR markers	Chromosome	No. of alleles	Allelic richness [a]	Gene diversity	PIC [b]
RMS1107	1R	9	3.64	0.48	0.45
RMS1280	1R	4	2.18	0.17	0.16
RMS1303	1R	7	2.34	0.17	0.17
SCM247	1R	5	2.61	0.28	0.26
SCM266	1R	3	2.04	0.13	0.12
RMS1138	2R	7	3.59	0.37	0.35
RMS1230	2R	6	4.08	0.50	0.47
RMS1238	2R	6	3.52	0.42	0.39
SCM276	2R	3	2.58	0.30	0.27
SCM290	2R	5	3.23	0.45	0.41
SCM294	3R	3	2.14	0.41	0.33
RMS1028	3R	10	4.26	0.58	0.54
RMS1254	3R	7	3.55	0.40	0.37
RMS1261	3R	11	3.77	0.28	0.28
RMS1323	3R	4	1.67	0.08	0.08
RMS1007	4R	25	6.68	0.65	0.63
RMS1026	4R	5	2.16	0.21	0.19
RMS1181	4R	2	2.00	0.41	0.32
SCM047	4R	2	1.94	0.21	0.19
RMS1083	5R	18	5.98	0.57	0.55
RMS1205	5R	6	2.81	0.36	0.33
RMS1218	5R	4	3.42	0.47	0.43
RMS1237	5R	9	3.72	0.35	0.34
RMS1259	5R	8	4.49	0.58	0.54
RMS1278	5R	5	2.96	0.46	0.40
SCM260	5R	5	2.20	0.13	0.13
RMS1090	6R	7	3.24	0.50	0.44
RMS1121	6R	15	6.44	0.66	0.63
SCM107	6R	2	1.97	0.25	0.22
SCM214	6R	9	5.22	0.66	0.63
RMS1012	7R	15	5.79	0.58	0.56
RMS1018	7R	12	5.34	0.53	0.51
RMS1187	7R	3	2.00	0.34	0.28
RMS1188	7R	4	3.17	0.58	0.52
RMS1197	7R	6	2.82	0.31	0.29
SCM063	7R	4	2.93	0.57	0.49
SCM322	7R	5	4.06	0.69	0.65
Mean		7.05	3.42	0.41(0.35-0.46)[c]	0.38(0.33-0.43)[c]

[a] Allelic richness is a measure of the number of alleles independent of sample size, a higher value means higher genetic diversity.

[b] PIC: Polymorphic information content, a higher value means higher genetic diversity.

[c] 95% confidence intervals obtained by 10,000 bootstraps across loci.
