# Supplementary material for: High levels of nucleotide diversity and fast decline of linkage disequilibrium in rye (Secale cereale L.) genes involved in frost response
Source: BMC Plant Biol. 2011 Jan 10;11:6. doi: 10.1186/1471-2229-11-6 (PMC3032657; doi:10.1186/1471-2229-11-6)
Supplement: Additional file 4 — Analysis of molecular variance (AMOVA) based on 37 SSR markers. [file 1471-2229-11-6-S4.PDF]

Additional file 4: Analysis of molecular variance (AMOVA) based on 37 SSR markers.

Source	Df	Sum of squares	Variance components	Percentage of total genetic variance
**Among populations**	4	235,198	1.30***	13.3%
**Among individuals within populations**	196	1,801,269	8.50***	86.7%
**Total**	200	2,036,468	9.80	100%

ns: not significant.

*** indicates significance (*P*<0.001), obtained from 15,000 permutations.
