# Supplementary material for: High levels of nucleotide diversity and fast decline of linkage disequilibrium in rye (Secale cereale L.) genes involved in frost response
Source: BMC Plant Biol. 2011 Jan 10;11:6. doi: 10.1186/1471-2229-11-6 (PMC3032657; doi:10.1186/1471-2229-11-6)
Supplement: Additional file 5 — Scatterplots of pairwise distances and LD. [file 1471-2229-11-6-S5.PDF]

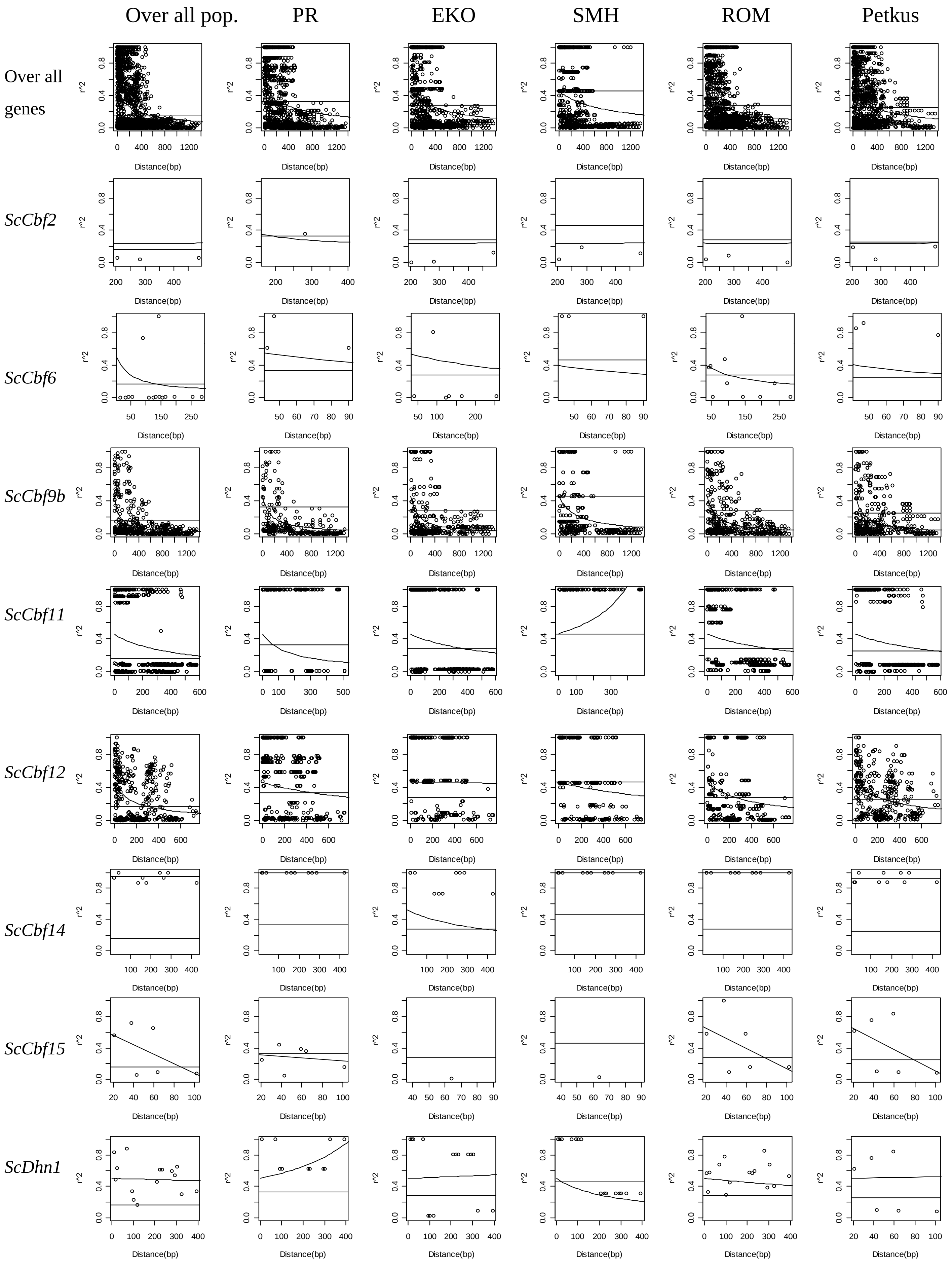
Over all pop.
PR
EKO
SMH
ROM
Petkus
Over all genes
ScCbf2
ScCbf6
ScCbf9b
ScCbf11
ScCbf12
ScCbf14
ScCbf15
ScDhn1
r^2
Distance(bp)

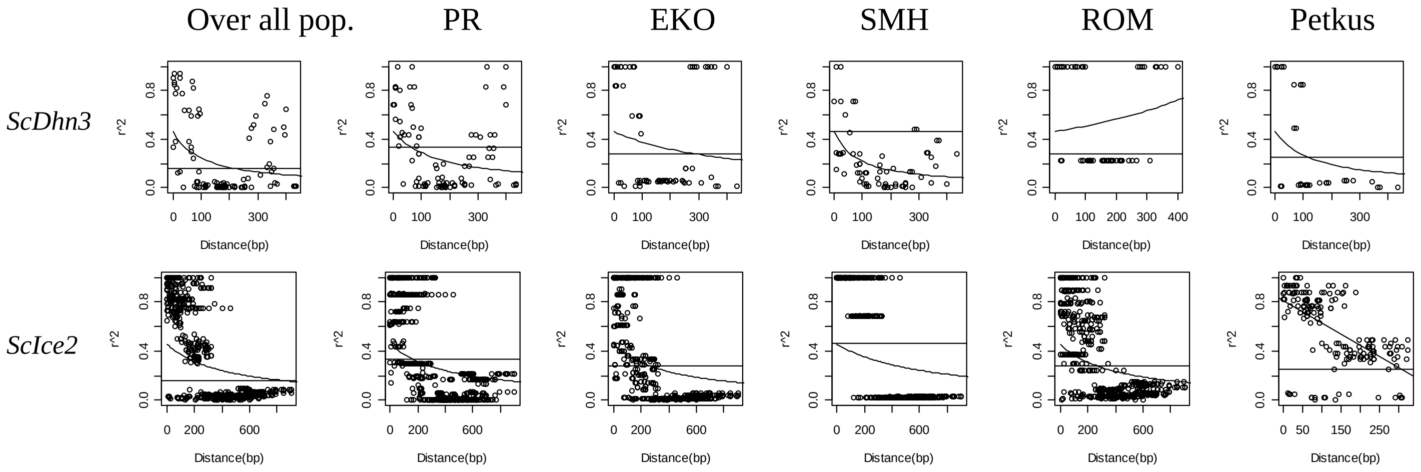

Additional file 5: Scatterplots of pairwise distances and LD estimated by r^2 between all SNPs (MAF>5%) in eleven candidate genes (for all and for individual genes) in five populations (for all and for individual populations). The non-linear fitting curve of the mutation-recombination-drift model is shown. Thresholds for LD are indicated by horizontal solid lines which are 0.16 (over all populations) , 0.33 (PR), 0.28 (EKO), 0.46 (SMH) , 0.28 (ROM), and 0.25(Petkus).
